# Injury of the cingulum in patients with putaminal hemorrhage: a diffusion tensor tractography study

**DOI:** 10.3389/fnhum.2014.00366

**Published:** 2014-05-30

**Authors:** Hyeok Gyu Kwon, Byung Yeon Choi, Seong Ho Kim, Chul Hoon Chang, Young Jin Jung, Han Do Lee, Sung Ho Jang

**Affiliations:** ^1^Department of Physical Medicine and Rehabilitation, College of Medicine, Yeungnam UniversityDaegu, South Korea; ^2^Department of Neurosurgery, College of Medicine, Yeungnam UniversityDaegu, South Korea

**Keywords:** cingulum, putaminal hemorrhage, cognitive function, diffusion tensor tractography, location of hematoma

## Abstract

**Objectives:** Little is known about the pathophysiological mechanisms of cognitive impairment in patients with putaminal hemorrhage (PH). Using diffusion tensor tractography, we investigated injury of the cingulum in patients with PH.

**Methods:** We recruited 63 patients with PH, who were classified according to three groups, based on integrity of the cingulum to the lower portion of the genu of the corpus callosum: group A; preserved integrity, group B; discontinuation of integrity in the affected hemisphere, and group C; discontinuation of integrity in both hemispheres.

**Results:** Thirty four patients (54.0%) belonged to group A, 16 patients (25.4%) to group B, and the remaining 13 patients (20.6%) to group C. Regarding the Mini-Mental State Examination, significant differences were observed between group A and group C, and between group B and group C without significant difference between group A and group B (*p* < 0.05). In terms of the volume of hematoma, significant differences were observed among the three groups (*p* < 0.05). Regarding the most anterior point of the hematoma, significant differences were observed between group A and groups B and C (*p* < 0.05); in contrast, regarding the most point of hematoma, significant differences were observed between group C and groups A and B, respectively (*p* < 0.05).

**Conclusion:** We found that the anterior cingulum is vulnerable to PH. Therefore, our results suggest the necessity for evaluation of the cingulum in patients with PH particularly if the hematoma is large or close to the anterior margin or midline of the brain.

## INTRODUCTION

The putamen is a common site of spontaneous intracerebral hemorrhage (ICH; Inagawa et al., 2003; Ghetti, 2012). Putaminal hemorrhage (PH) shows a large range of clinical manifestations because it can involve many important neural tracts for somatosensory-motor function, language, and visuo-perception (Mendoza and Foundas, 2007; Ghetti, 2012; Jang, 2013; Martino et al., 2013; Seo et al., 2013). Therefore, the typical clinical manifestations of PH include motor weakness, somatosensory deficit, homonymous hemianopsia, consciousness impairment, aphasia, and spatial neglect (Bogousslavsky and Caplan, 2001; Ghetti, 2012). By contrast, significantly fewer studies have reported on cognitive impairment in patients with PH (Hochstenbach et al., 1998; Shu et al., 2002; Su et al., 2007). In addition, little is known about the pathophysiological mechanisms of cognitive impairment in patients with PH (Yeo et al., 2012).

The cingulum, the long neural tract extending, between the orbitofrontal, parietal, and medial temporal lobes (Goldman-Rakic et al., 1984; Mendoza and Foundas, 2007), is known to be involved in various cognitive functions, including attention, memory, learning, motivation, and emotion (Vogt et al., 1992; Bush et al., 2000; Botvinick et al., 2004). In particular, it is involved in memory function because the cingulum contains the medial cholinergic pathway, which originates from the nucleus basalis of Meynert (Ch 4) in the basal forebrain and obtains cholinergic innervation from two cholinergic nuclei (the medial septal nucleus; Ch1, the vertical nucleus of the diagonal band; Ch2) in the septal region (Selden et al., 1998; Lucas-Meunier et al., 2003; Nieuwenhuys et al., 2008). Therefore, injury of the cingulum can cause cognitive impairment, including memory impairment.

Exact diagnosis of injury of the cingulum has been difficult using conventional brain CT or MRI because these imaging techniques cannot discriminate the cingulum from adjacent neural structures. However, recently developed diffusion tensor tractography (DTT), derived from diffusion tensor imaging (DTI), enables three dimensional reconstruction and evaluation of the architecture and integrity of the cingulum (Concha et al., 2005). Consequently, many studies have reported on injury of the cingulum in various brain pathologies using DTI (Sugiyama et al., 2009; Wilde et al., 2010, 2011; Wu et al., 2010; McCauley et al., 2011; Wang et al., 2011; Lee et al., 2012; Baek et al., 2013). However, no study on injury of the cingulum in patients with PH has been reported. We hypothesized that injury of the cingulum can occur as a result of PH, despite its location far from the putamen.

In the current study, using DTT, we attempted to investigate injury of the cingulum in patients with PH.

## MATERIALS AND METHODS

### SUBJECTS

We recruited 63 patients (male: 39, female: 24, mean age: 53.5 ± 10.1 years, range: 33~ 67 years) who had been admitted for rehabilitation to the rehabilitation department of a university hospital for this study. Inclusion criteria for patients were as follows: (1) first ever stroke, (2) a hematoma located primarily in the lentiform nucleus of the basal ganglia, (3) DTI scan performed at early stage (between 1 and 5 weeks) after onset, and (4) no hydrocephalus, subarachnoid hemorrhage, or intraventricular hemorrhage. This study was conducted retrospectively and the study protocol was approved by the Institutional Review Board of a university hospital.

### CLINICAL EVALUATION

Cognitive function of the patients was evaluated using the Mini-Mental State Examination (MMSE) at the same time that DTI scanning was performed. The reliability and validity of the MMSE have been well established (Dick et al., 1984).

### MEASUREMENT OF THE VOLUME OF HEMATOMA AND HEMATOMA LOCATION OF THE MOST ANTERIOR AND MEDIAL POINT OF THE LESION

Volume of hematoma was measured on T2-weighted MRI images using a picture-archived communication system (PACS, Marotech, Korea). We measured maximum width (*X*), length (*Y*), and height (*Z*) of the lesion at the level where hemorrhage of the lentiform nucleus of the basal ganglia could be clearly observed (Kwak et al., 1983). Volume of hematoma was calculated according to the formula:

(1) $$Volume\,of\,hematoma\,\left(mV\right)\,=\frac{4}{3}\times \frac{1}{16}\,\times \pi \times X(cm)\times Y(cm)\times Z\left(cm\right)$$

For the most anterior point of a hematoma, we measured the distance between the most anterior margin of the brain and the most anterior point of the hematoma in the antero-posterior direction, and then divided by the distance between the most anterior margin and posterior margin of the brain in the antero-posterior direction. Regarding the most medial point of a hematoma, we measured the distance between the midline and the most medial point of a hematoma in the medio-lateral direction, and then divided by the distance between the midline and the most lateral margin of the brain in the medio-lateral direction.

### DIFFUSION TENSOR TRACTOGRAPHY

A six-channel head coil on a 1.5 T Philips Gyroscan Intera (Philips, Ltd., Best, The Netherlands) with single-shot echo-planar imaging was used for acquisition of DTI data at a mean of 2.6 weeks (range: 1 ~ 5 weeks). For each of the 32 non-collinear diffusion sensitizing gradients, we acquired 70 contiguous slices parallel to the anterior commissure-posterior commissure line. Imaging parameters were as follows: acquisition matrix = 96 × 96; reconstructed to matrix = 192 × 192; field of view = 240 mm × 240 mm; TR = 10,726 ms; TE = 76 ms; parallel imaging reduction factor (SENSE factor) = 2; EPI factor = 49; *b* = 1000 s/mm^2^; NEX = 1; and a slice thickness of 2.5 mm. Removal of eddy current-induced image distortions was performed at the Oxford Centre for functional magnetic resonance imaging of brain software library (FSL; www.fmrib.ox.ac.uk/fsl) using affine multi-scale two-dimensional registration (Smith et al., 2004). Reconstruction of the cingulum was performed using DTI-Studio software (CMRM, Johns Hopkins Medical Institute, Baltimore, MD, USA; Jiang et al., 2006). Before fiber tracking, calculation of DTI data was performed automatically using DTI-Studio software (Jiang et al., 2006). The cingulum was determined by selection of fibers passing through two regions of interest (ROIs) based on the fiber assignment continuous tracking (FACT) algorithm (Concha et al., 2005). The first ROI was drawn at the middle portion of the cingulum (green color) on the coronal image with the color map (blue color: superioinferior orientation, red color: mediolateral orientation, green color: anteroposterior orientation). The second ROI was given at the posterior portion of the cingulum (green color) on the coronal image with the color map (Concha et al., 2005). Fiber tracking was started at any seed voxel with a fractional anisotropy (FA) >0.18 and ended at a voxel with a FA of <0.18 and a tract turning-angle of <60°.

For measurement of intra- and inter-observer reliability, random analyses of the data was performed by two evaluators (Kwon and Lee) who were blinded to the other evaluator's data. The consistency rate of analyses with three tract turning angles by two evaluators were identical for 123 out of 126 hemispheres (97.6%), and two sets of analyses performed by one analyzer (Kwon) were identical for 126 out of 126hemispheres (100%).

**FIGURE 1:**
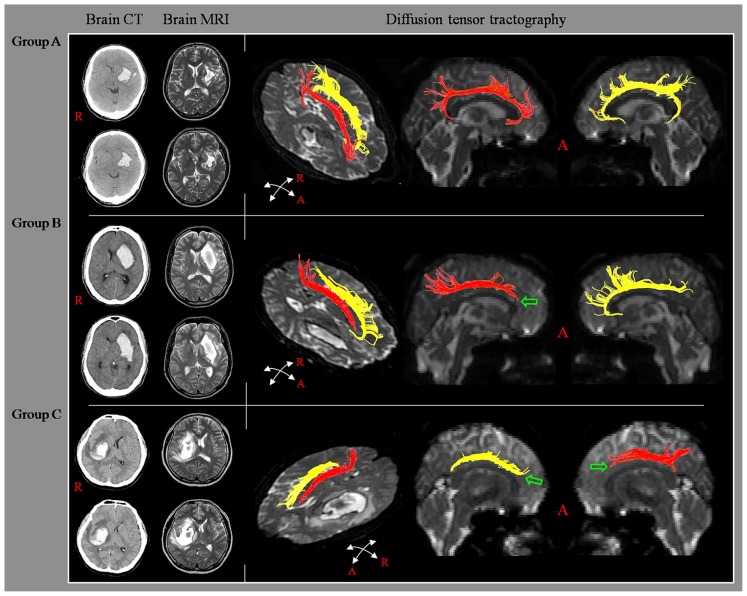
**Classification of diffusion tensor tractography (DTT) for the cingulum.** Diffusion tensor tractography: group A; both sides of the cingulum showed intact continuity to the lower portion of the genu of the corpus callosum, group B; either side of the cingulum showed a discontinuation to the lower portion of the genu of the corpus callosum, and group C; both sides of the cingulum showed discontinuation to the lower portion of the genu of the corpus callosum.

According to findings for the cingulum on DTT, the patients were classified according to three groups, based on integrity of the cingulum to the lower portion of the genu of the corpus callosum: group A; both sides of the cingulum showed intact continuity to the lower portion of the genu of the corpus callosum, group B; either side of the cingulum showed a discontinuation to the lower portion of the genu of the corpus callosum, and group C; both sides of the cingulum showed discontinuation to the lower portion of the genu of the corpus callosum (**Figure 1**).

### STATISTICAL ANALYSIS

One-way analysis of variance (ANOVA) was performed for determination of differences in the MMSE, volume of hematoma, and hematoma location of the most anterior and medial point between the three groups. When a significant difference was detected in the ANOVA between three groups, least significant difference (LSD) *post hoc* test was performed for determination of the differences in the MMSE, volume of hematoma, and hematoma location of the most anterior and medial point between the three groups. In addition, an independent *t*-test was performed for determination of differences in the MMSE between lesion side of right and left hemisphere. The significant level of the *p* value was set at 0.05.

## RESULTS

A summary of the demographic data for the three groups is shown in **Table 1**. Among 63 patients, 34 patients belonged to group A [54.0%, male: 18, female: 16, mean age: 52.6 ± 9.6, lesion side (Rt/Lt): 16/18], 16 patients to group B (25.4%, male: 12, female: 4, mean age: 51.8 ± 10.0, lesion side: 10/6), and the remaining 13 patients to group C (20.6%, male: 9, female: 4, mean age: 56.3 ± 9.0, lesion side: 6/7). According to the results, 46.0% (29 of 63 patients) and 20.6% (13 of 63 patients) showed discontinuation of the lower portion of the genu of the corpus callosum in the affected hemisphere and both hemispheres, respectively.

**Table 1:** Demographic data of the three groups.

Variables	Group A	Group B	Group C
Patients, *n* (%)	34 (54.0)	16 (25.4)	13 (20.6)
Age (year)	52.6 (9.6)	51.8 (10.0)	56.3 (9.0)
Sex, male/female	18/16	12/4	9/4
Lesion side (Rt/Lt)	16/18	10/6	6/7
Mean days to DTT or duration from onset, days	18.8 (8.2)	20.7 (12.2)	17.8 (7.5)

*Values represent mean (±standard deviation); DTT, diffusion tensor tractography.*

A summary of the MMSE, volume of hematoma, and hematoma location of the most anterior and medial point for the three groups is shown in **Table 2**. Regarding the results of ANOVA analysis, significant differences were observed between the three groups, as follows: MMSE (*F* = 17.035, *p* < 1 × 10e-4), volume of hematoma (*F* = 16.915, *p* < 1 × 10e-4), hematoma location of the most anterior point (*F* = 7.748, *p* = 0.001), and hematoma location of the most medial point (*F* = 11.559, *p* < 1 × 10e-4). In detail, regarding the *post hoc* test of the MMSE, significant differences were observed between group A and group C, and between group B and group C (*p* < 0.05). However, no significant difference was observed between group A and group B (*p* > 0.05). Significant differences in the volume of hematoma were observed among the three groups (*p* < 0.05). The most anterior point of hematoma for groups A, B, and C was located at 31.75, 25.60, and 26.22% posteriorly from the most anterior margin of the brain, respectively. The most medial point of hematoma for groups A, B, and C was located at 27.68, 25.37, and 18.90% laterally from the midline, respectively. Regarding the most anterior point of hematoma, significant differences were observed between group A and groups B and C (*p* < 0.05), however, no significant difference was observed between group B and group C (*p* > 0.05). Regarding the most medial point of hematoma, significant differences were observed between group C and groups A and B, respectively (*p* < 0.05), however, no significant difference was observed between group A and group B (*p* > 0.05). On the other hand, MMSE for the lesion side of the right and left hemisphere were 24.4 and 23.1, respectively. No significant differences were observed between the lesion side of the right and left hemispheres (*p >* 0.05)

**Table 2:** Clinical and hematoma data according to groups of patients.

Variables		Group			*p* Value		
		A	B	C	A–B	A–C	B–C
MMSE		26.24 (3.7)	24.69 (3.1)	16.08 (9.7)	0.347	<1 × 10e-4^*^	<1 × 10e-4^*^
Hematoma	Volume (mV)	6.93 (3.5)	10.69 (4.7)	13.75 (3.2)	0.002^*^	<1 × 10e-4^*^	0.034^*^
	Anterior point (%)	31.75 (7.1)	25.60 (4.9)	26.22 (2.4)	0.001^*^	0.006^*^	0.780
	Medial point (%)	27.68 (5.9)	25.37 (5.3)	18.90 (4.9)	0.180	<1 × 10e-4^*^	0.003^*^

Values represent mean (±standard deviation), MMSE: Mini-Mental State Examination, anterior point: posteriorly the most anterior point from the most anterior margin of the brain, medial point: laterally the most medial point from the midline, lesion location of the most anterior and medial point of the lesion represented the percentage as whole length and width of brain. *p < 0.05.

## DISCUSSION

In this study, using DTT, we investigated injury of the cingulum in patients with PH, and observed the following results. First, regarding the incidence of injury of the cingulum, 25.4% of patients with PH showed injury of the anterior cingulum in the affected hemisphere and 20.6% of patients showed injury of the anterior cingulum in both the unaffected hemisphere and the affected hemisphere. As a result, 46% of patients with PH showed an injury of the anterior cingulum, at least in the affected hemisphere. Second, significant differences in volume of hematoma were observed among the three groups, in the following order: group C > group B > group A. These results indicate that injury of the anterior cingulum was affected by the volume of hematoma. Third, the MMSE was lower in patients with injury of the bilateral cingulum than in patients with injury of the cingulum only in the affected hemisphere or without injury of the cingulum. This result appears to be attributed to the characteristics of the cingulum, which obtains cholinergic innervations from three cholinergic nuclei (the medial septal nucleus; Ch1, the vertical nucleus of the diagonal band; Ch2, and the nucleus basalis of Meynert; Ch 4; Selden et al., 1998; Lucas-Meunier et al., 2003; Nieuwenhuys et al., 2008). Fourth, the most anterior and medial points of hematoma: The most anterior point of hematoma was affected by injury of the cingulum in the affected hemisphere; in contrast, the most medial point of hematoma was affected by injury of the cingulum in the unaffected hemisphere.

Many studies have reported on the possible pathophysiological mechanisms of cognitive impairment in patients with basal ganglia pathology and the neural tracts related to cognition (Alexander et al., 1986; Herrero et al., 2002; Rektor et al., 2004; Jang and Yeo, 2013; Martino et al., 2013). However, only a few studies have reported on the pathophysiological mechanism of cognitive impairment in patients with PH (Yeo et al., 2012). Yeo et al. (2012) who investigated the incidence of fornix injury in 58 consecutive patients with PH, found that 6 (10.7%) of 58 patients showed complete disruption of the fornix body on DTT. Therefore, to the best of our knowledge, this is the first study to investigate injury of the cingulum in patients with PH.

Since introduction of DTI, many studies have investigated injury of the cingulum in various brain pathologies, including subarachnoid hemorrhage, traumatic brain injury, and hypoxic ischemic brain injury (Sugiyama et al., 2009; Wilde et al., 2010, 2011; Wu et al., 2010; McCauley et al., 2011; Wang et al., 2011; Hong et al., 2012; Lee et al., 2012; Baek et al., 2013; Sanjuan et al., 2013; Zheng et al., 2013). Most of these studies investigated injury of the cingulum using DTI parameters [Sugiyama et al., 2009; Wilde et al., 2010, 2011; Wu et al., 2010; McCauley et al., 2011; Wang et al., 2011; Hong et al., 2012; Lee et al., 2012; Sanjuan et al., 2013; Zheng et al., 2013 (Epub ahead of print)]. Only a few studies have reported that discontinuation of the anterior cingulum was related to cognitive dysfunction following brain injury (Baek et al., 2013). Baek et al. (2013), who investigated the relation between integrity of the anterior cingulum and cognitive impairment in 35 patients with traumatic brain injury, reported lower cognitive function in terms of the intelligence quotient of the Wechsler Intelligence Scale and Memory Assessment Scale in patients with injury of the bilateral anterior cingulum than in patients with injury of the unilateral anterior cingulum or patients without injury of the cingulum. Therefore, our results showing lower MMSE in patients with injury of the bilateral cingulum than in patients with injury of the cingulum only in the affected hemisphere or without injury of the cingulum appear to coincide with the results of this study.

In conclusion, we investigated injury of the cingulum in patients with PH and found that 25.4% of patients with PH showed injury of the anterior cingulum in the affected hemisphere and 20.6% of patients showed injury in both hemispheres. As a result, 46% of patients with PH showed an injury of the anterior cingulum, at least in the affected hemisphere. The anterior cingulum appears to be affected by the size of the hematoma. In addition, injury of the anterior cingulum in the affected hemisphere was affected by the anterior invasion of hematoma and injury of the anterior cingulum in the unaffected hemisphere was affected by the medial invasion of hematoma. Our results indicate that the anterior cingulum is vulnerable to PH. Therefore, we suggest the necessity for evaluation of the cingulum in patients with PH, particularly when the hematoma is large or close to the anterior margin or midline of the brain. Several limitations should be considered in interpretation of this study. First, we recruited patients among those with PH who had been admitted for rehabilitation. This suggests the possibility that we recruited patients with severe clinical manifestations among all patients with PH. Second, because this study was conducted retrospectively, we were not able to obtain detailed neuropsychological data, except for MMSE. Therefore, conduct of further prospective studies involving detailed neuropsychological data would be necessary. Third, ICH, particularly large hemorrhage, might form artifacts and affect our results. Finally, DTT may underestimate fiber tracts due to peri-hematomal edema or previous undetected traumatic brain injury. In addition, fiber complexity and crossing fiber effect can prevent reflection of the fiber tracts (Wedeen et al., 2008; Yamada et al., 2009). Therefore, conduct of further studies in order to overcome these limitations of DTI should be encouraged.

## Conflict of Interest Statement

The authors declare that the research was conducted in the absence of any commercial or financial relationships that could be construed as a potential conflict of interest.
